# The global prevalence of myocardial infarction: a systematic review and meta-analysis

**DOI:** 10.1186/s12872-023-03231-w

**Published:** 2023-04-22

**Authors:** Nader Salari, Fatemeh Morddarvanjoghi, Amir Abdolmaleki, Shabnam Rasoulpoor, Ali Asghar Khaleghi, Leila Afshar Hezarkhani, Shamarina Shohaimi, Masoud Mohammadi

**Affiliations:** 1 Department of Biostatistics, School of Health, Kermanshah University of Medical Sciences, Kermanshah, Iran; 2 Sleep Disorders Research Center, Kermanshah University of Medical Sciences, Kermanshah, Iran; 3 Student Research Committee, Kermanshah University of Medical Sciences, Kermanshah, Iran; 4 Department of Operating Room, Nahavand School of Allied Medical Sciences, Hamadan University of Medical Sciences, Hamadan, Iran; 5 Department of Psychiatric Nursing, Miandoab School of Nursing, Urmia University of Medical Sciences, Urmia, Iran; 6 Cellular and Molecular Research Center, Gerash University of Medical Sciences, Gerash, Iran; 7 Neuroscience Research Center, Health Technology Institute, Kermanshah University of Medical Sciences, Kermanshah, Iran; 8 Department of Biology, Faculty of Science, University Putra Malaysia, Serdang, Selangor Malaysia

**Keywords:** Prevalence, Myocardial infarction, MI, Heart attack

## Abstract

**Background:**

Myocardial infarction (MI) is one of the life-threatening coronary-associated pathologies characterized by sudden cardiac death. The provision of complete insight into MI complications along with designing a preventive program against MI seems necessary.

**Methods:**

Various databases (PubMed, Web of Science, ScienceDirect, Scopus, Embase, and Google scholar search engine) were hired for comprehensive searching. The keywords of “Prevalence”, “Outbreak”, “Burden”, “Myocardial Infarction”, “Myocardial Infarct”, and “Heart Attack” were hired with no time/language restrictions. Collected data were imported into the information management software (EndNote v.8x). Also, citations of all relevant articles were screened manually. The search was updated on 2022.9.13 prior to the publication.

**Results:**

Twenty-two eligible studies with a sample size of 2,982,6717 individuals (< 60 years) were included for data analysis. The global prevalence of MI in individuals < 60 years was found 3.8%. Also, following the assessment of 20 eligible investigations with a sample size of 5,071,185 individuals (> 60 years), this value was detected at 9.5%.

**Conclusion:**

Due to the accelerated rate of MI prevalence in older ages, precise attention by patients regarding the complications of MI seems critical. Thus, determination of preventive planning along with the application of safe treatment methods is critical.

## Background

Myocardial Infarction (MI) is one of the life-threatening coronary events with SCD [1] and the most severe clinical presentation of coronary artery disease (CAD) [2]. This pathology is divided into two categories of ST-elevation MI (STE-MI) and non-ST-elevation MI (NSTE-MI). Since unstable angina is the imminent background for MI, it is also considered an acute coronary syndrome (ACS) status [3].

More than 3 million individuals develop STE-MI each year, and more than 4 million people represent STE-MI pathology. Although MI is mainly detected in developed countries, it is also detected commonly in developing countries [4–7]. In a published study with 19,781 CAD patients, the MI prevalence was found 23.3% [8]. In recent years, a considerable decreasing trend in STE-MI incidence was detected in European countries and the United States [9, 10].

MI is the main cause of human death, globally [11]. Although the global rate of MI-associated mortality was totally decreased, the incidence of heart failure (HF) is at a high level [12]. The mortality and morbidity rates are high in MI-related HF [13, 14]. HF induces detrimental impacts on the healthcare systems of the United States, affecting 6 million individuals, 300,000 deaths per year, and approximately $40 billion in costs [15]. Also, the economic impact of MI is at a high rate. In 2010, more than 1.1 million hospitalizations following MI attacks were reported in the United States, with an estimated direct cost of $450 billion [16]. Body weakness is a common complication in cardiovascular diseases and is also a common syndrome among the elderly causing weight loss, fatigue, physical manipulation, decreased walking speed, and low body activity [17]. Obesity, sedentary lifestyle, hypertriglyceridemia, or inflammation markers (such as high-sensitivity C-reactive protein [hs-CRP]), are mostly independent cardiovascular (CV) risk factors associated with insulin [18]. Various published articles represented a general increase in the prevalence of cardiovascular risk factors (especially diabetes, cholesterol and obesity, and even smoking) [19–22]. In MI patients < 55 years, smoking was found a unique cardiovascular risk factor in 80% of cases [23].

The present systematic review and meta-analysis study seems beneficial for health system policymakers requiring the prevalence of MI patients during the allocation of health care resources. We believe that elimination of the complications and reduction in mortality rate need comprehensive assessment approaches.

## Methods

In this study, the primary search was conducted on June 6, 2022. Databases of PubMed, Web of Science, ScienceDirect, Scopus, Embase, and Google scholar search engine were hired for definition of searching strategy. Also, the main keywords of “Prevalence”, “Outbreak”, “Burden”, “Myocardial Infarction”, “Myocardial Infarct”, and “Heart Attack” were used for comprehensive searching with no time and language-associated restrictions. Following paper selection, the related citations were imported to the information management software (EndNote v.8x). Finally, in order to secondary screening, all citations of the collected articles were reviewed manually. The searching was also updated on September 13, 2022.

### Inclusion and exclusion criteria

All gathered studies reporting the MI prevalence, available full texts, and studies with sufficient data (number of samples, percentage of MI prevalence) were totally included in this study. Also, case–control studies, cohort investigations, case series, case reports, reviews, repetitive papers, studies with insufficient data, papers with unavailable full texts, and conference studies were excluded.

### Study selection

The Endnote software (v. X8) was hired to organize the selected studies. Duplicate studies were detected and merged together. In primary screening, irrelevant studies were removed following assessment of the titles and abstracts. Then, the full texts of the remaining articles were screened according to the inclusion and exclusion criteria. All screening protocols were conducted by two independent authors in order to accelerate the credibility index and inhibit the potential searching bias. Corresponding author was also responsible for the management of possible disagreements among the researchers. Finally, 33 studies were included for quality control assessment.

### Quality control assessment

For validation and the quality control assessment, an observational study-associated checklist (The Strengthening the Reporting of Observational Studies in Epidemiology checklist (STROBE)) was used. This STROBE checklist consisted of six assessment scales of Title, Abstract, Introduction, Methodology, Results, and Discussion with 32 evaluation items including Title, Problem Statement, Study Objectives, Type of Study, Statistical Population, Sampling Method, Appropriate Sample Size Determination, Variables Definition, and the Procedures, Data Collection Tools, Statistical Analysis Methods and Findings. The article with STROBE scoring ≥ 16 was considered good and moderate (included in the study), and articles < 16 were poor quality (excluded from the study).

### Data extraction

The eligible data were extracted by two researchers based on the previously prepared checklist (containing the Author's name, Year of publication, Research region, Sample size, Disease prevalence, and Age).

### Data analysis

The heterogeneity of the studies was assessed using I^2^ test. Also, the Egger test was used for publication bias assessment. All statistical analysis was applied in Comprehensive Meta-Analysis software (Version 2).

## Results

**Fig. 1:**
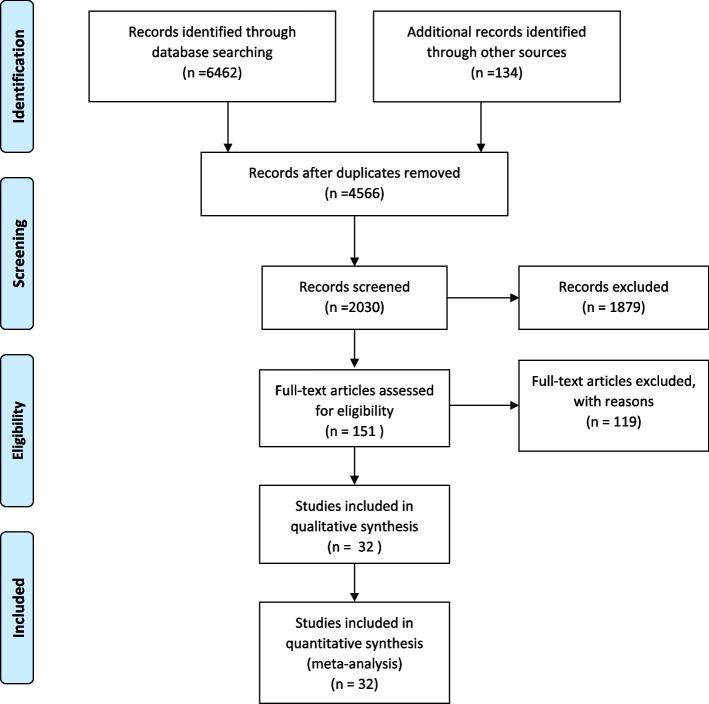
Reviewing, screening and extracting articles based on PRISMA process

Whole eligible data (6462 studies systematically and 134 investigations manually) regarding the prevalence of MI were collected based on the PRISMA guideline and categorized into two groups of individuals < 60 and ≥ 60 years. All the papers were imported into the information management software (EndNote v.X8). Among the total number of 6596 studies, 4566 duplicate investigations were detected and merged together. During the primary screening, the Title and Abstract of the remaining studies were assessed. Subsequently, 1879 investigations were excluded due to the irrelevant contents. Following the secondary screening, the full texts of the papers were assessed (118 studies were also excluded in this stage). Eligible collected papers were assessed based on the STROBE checklist, and the studies with poor-quality methodology were removed from the investigation. Finally, 32 high-quality papers were included for systematic review and meta-analysis study (Table 1) (Fig. 1).

**Table 1:** Studies obtained and information extracted from them

**Authors**	**Region**	**Year**	**Sample size**	**Number of patients**	**Prevalence**	**Age**	**Instruments**
Chow, C. M and et al. [24]	Canada	2005	3,318,117 4,118,589 4,746,631 5,077,402 3,637,171 2,396,167 1,744,169 749,088	975 2477 8216 36,183 99,768 131,361 172,095 85,474	0.0 0.1 0.2 0.7 2.7 5.5 9.9 11.4	12–19 20–29 30–39 40–49 50–59 60–69 70–79 80 ≤	Self-reported data
Assante, R and et al. [25]	Italy	2015	2420	758	31.3	36–14	Between January 2009 and December 2013, 2420 consecutive subjects (258 inmates and 2162 non-inmates) with suspected or known coronary artery disease underwent stress myocardial perfusion single-photon emission computed tomography (MPS) to our institution
Carrillo, X and et al. [26]	Spain	2011	479	58	12.1	49–38	questionnaire about cocaine use and frequency of use as well as a urine test for cocaine within 48–72 h of admission
Bosch, X and et al. [27]	Spain	2010	402 370 467	5 19 34	1.2 5.1 7.2	34–25 44–35 54–45	standard questionnaire
Bulow, B and et al. [28]	Sweden	2000	33	1	3.03	46–6	hypopituitary patients
Chung, E. H and et al. [29]	USA	2007	161	119	73.9	45–18	using data retrieved from the National Cardiovascular Data Registry at Lahey Clinic, who underwent cardiac catheterization for AMI from June 2001 to December 2004
Domingos, F and et al. [30]	Portugal	2011	23,349	443	1.9	45–18	The sample used in the fourth NHS was randomly selected from a mother sample used by the NIS for studies with families among residents in private households from a representative sample of households from the mainland and the autonomous regions of Azores and Madeira, using a system of stratification and systematic selection
Gikas, A and et al. [31]	Greece	2008	0 600 527 787 258 199	0 9 20 70 109 38	0 1.5 3.8 8.9 23.8 19.1	34–20 44–35 54–45 64–55 74–65 75 ≤	Self-reported data
Gisondi, P and et et al. [32]	Italy	2011	482	174	36.09	55–52	using a structured questionnaire
Ingelfinger, J. A and et al. [33]	Indian	1976	120	14	11.6	60–40	Twelve-lead electrocardiograms (ECG) were obtained in the postprandial state from 351 male and 350 female Pima Indians
Khan, H and et al. [34]	Texas	2022	1409	93	6.6	64 <	Hospital patient data for those with and without a history of MI were obtained from the Project FRONTIER database for rural West Texas counties
Kitamura, A and et al. [35]	Japan	2002	17,404	114	0.65	59–40	The surveyed population included all male employees aged 40 to 59 years who worked for eight industrial companies in Osaka, the second largest metropolitan city in Japan
Lampe, F. C and et al. [36]	UK	2001	3718 5617 5714 3655 1637 22,179	15 33 33 26 11 132	0.4 0.5 0.5 0.7 0.6 0.5	44–40 49–45 54–50 59–55 64–60 69–65	The prevalences of current angina symptoms and history of diagnosed CHD were ascertained by questionnaire in 1978–80, 1983–85, 1992, and 1996
Lautsch, D and et al. [37]	USA	2019	39,100 48,927	3501 6152	9 12.6	74–65 75 ≤	We included de-identified adult patients with T2DM with at least one encounter in the CPRD database between 1 January 2018 and 31 December 2018 in the analysis and extracted the full health records of these patients
McCullough, Peter A and et al. [38]	USA	2008	301 2857 5324 8837 8740 3185	1 20 58 179 211 99	0.4 0.8 1.3 2.4 3.2 4.9	19–18 29–20 39–30 49–40 59–50 64–60	Community volunteers completed surveys regarding past medical events and underwent blood pressure and laboratory testing
Okoth, K and et al. [39]	UK	2017	1,475,676	2440	0.16	50–16	A series of annual (1998–2017) cohort and cross-sectional studies were conducted to estimate incidence rates and prevalence in men and women aged 16–50
Otaki, Y and et al. [40]	USA	2013	1981	42	2.1	58–40	Coronary CT Angiography Evaluation for Clinical Outcomes: An International Multicenter Registry (CONFIRM) is an international, multicenter, observational registry of 27,125 consecutive patients who underwent ≥ 64–detector row CCTA for suspected CAD at 12 centers from 2003 to 2009
Sato, K and et al. [41]	Japan	2020	3485 2601 510	537 266 223	15.4 10.2 43.7	79–70 89–80 90 ≤	The Miyagi AMI Registry is a prospective, multicenter, and observational study
Shaper, A. G and et al. [42]	British	1984	1838 1898 1974 2025	31 62 102 133	1.7 3.3 5.2 6.6	44–40 49–45 54–50 59–55	The prevalence of ischaemic heart disease was determined by an administered questionnaire and electrocardiography in 7735 men aged 40–59 years drawn at random from general practices in 24 British towns
Zeller, T and et al. [43]	Germany	2014	15,340	1980	12.9	58–39	High-sensitivity assayed troponin I was measured in the Scottish Heart Health Extended Cohort (*n* = 15 340) with 2171 cardiovascular events (including acute coronary heart disease and probable ischaemic strokes), 714 coronary deaths (25% of all deaths), 1980 myocardial infarctions, and 797 strokes of all kinds during an average of 20 years follow-up
Zeidan, R. K and et al. [44]	Lebanon	2016	506 351 234 270	15 26 16 25	2.9 7.3 7 9.2	50–40 60–50 70–60 70 ≤	We carried out a cross-sectional study using a multistage cluster sample across Lebanon. We interviewed residents aged 40 years and older using a questionnaire that captured the presence of CHDs and their risk factors (RFs)
Yoon, S. S and et al. [45]	Maryland	2012	3598	76 317	2.1 8.8	40–59 60 ≤	A total of 21,472 adults aged ≥ 40 years from the 2001–2012 National Health and Nutrition Examination Survey were included in the analysis. The analysis was conducted in 2015
Valentine, R. J and et al. [46]	America	1994	59	17	28.8	46–36	We studied the peripheral and coronary arterial circulations of 59 consecutive male military veterans diagnosed with premature peripheral vascular disease (age of onset :::;45 years) affecting the lower extremity
Schelbert, E. B and et al. [47]	ICELAND	2012	936	248	26.4	81–72	From a community-dwelling cohort of older individuals in Iceland, data for 936 participants aged 67 to 93 years were analyzed, including 670 who were randomly selected and 266 with diabetes
Kumar, A and et al. [48]	USA	2008	3224	368	11.4	78–66	Cardiovascular Health Study participants free of both clinical cardiovascular disease and major ECG abnormalities were included
Bahrmann, P and et al. [49]	Germany	2013	302	38	12.5	86–74	An emergency department (ED) of a city hospital covering a population of approximately 1 million in Germany Participants: A total of 332 consecutive unselected patients were recruited
Bethel, M. A and e et al. [50]	U.K	2017	2004	823	41.1	75 ≤	was a randomized, double-blind, placebo-controlled trial
Cauley, J. A and et al. [51]	USA	2016	5876	820	13.9	83–67	we performed a prospective study of 5994 men, primarily white, age 65 + years recruited at six US clinical centers
de la Torre Hernandez, J. M and et al. [52]	Spain	2017	3576	385	10.7	85–76	A 31-center registry of consecutive patients older than 75 years treated with primary angioplasty. Clinical and procedural data were collected, and the patients underwent clinical follow-up
Golledge, J and et al. [53]	Australia	2014	11,742	1711	14.5	76–67	A risk factor questionnaire which contained a question about salt intake was included as part of a population screening study for AAA in 11,742 older men. AAA presence was assessed by abdominal ultrasound imaging using a reproducible protocol
Ikeda, Y and et al. [54]	Japan	2014	14,464	74	0.5	76–67	The Japanese Primary Prevention Project (JPPP) was a multicenter, open-label, randomized, parallel-group trial. Patients (*N* = 14 464) were aged 60 to 85 years, presenting with hypertension, dyslipidemia, or diabetes mellitus recruited by primary care physicians at 1007 clinics in Japan between March 2005 and June 2007, and were followed up for up to 6.5 years, with last follow-up in May 2012
Teo, K. K and et al. [55]	Canada	2009	904	326	37	65 ≤	We conducted a pre-specified analysis of outcomes in stable CAD patients stratified by age and randomized to PCI OMT or OMT alone in the COURAGE (Clinical Outcomes Utilizing Revascularization and Aggressive druG Evaluation) trial

**Fig. 2:**
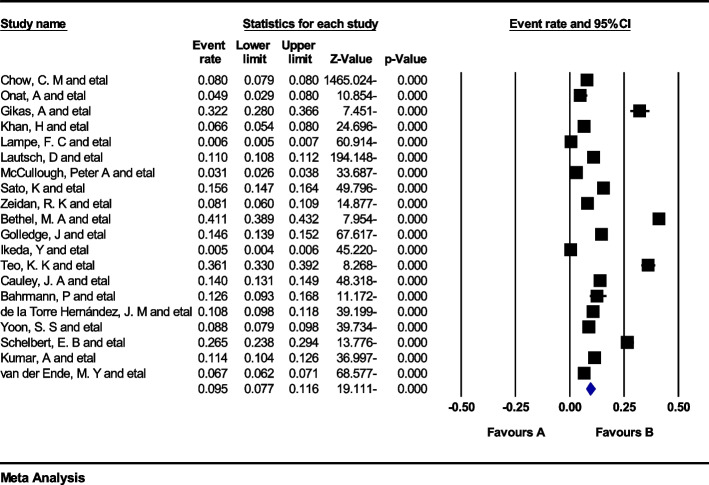
Forest plot representing the global prevalence of myocardial infarction in age group > 60 years based on the random effects model

**Fig. 3:**
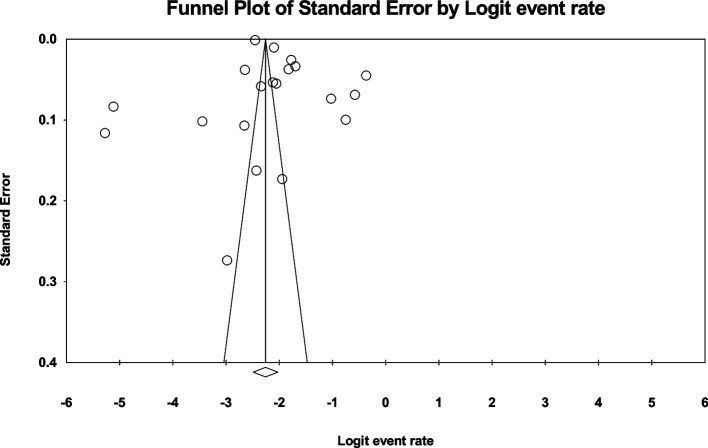
Funnel plot representing the distribution bias of eligible collected studies

**Fig. 4:**
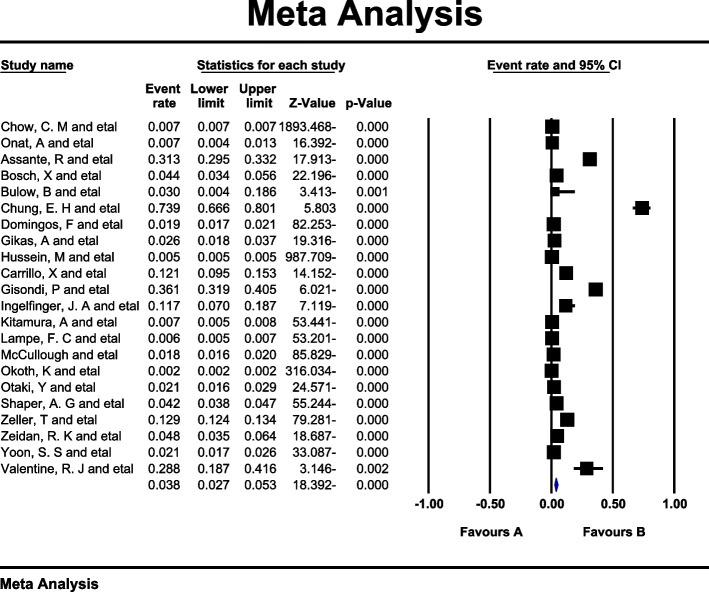
Forest plot representing the global prevalence of myocardial infarction in the age group < 60 years (random effect model)

**Fig. 5:**
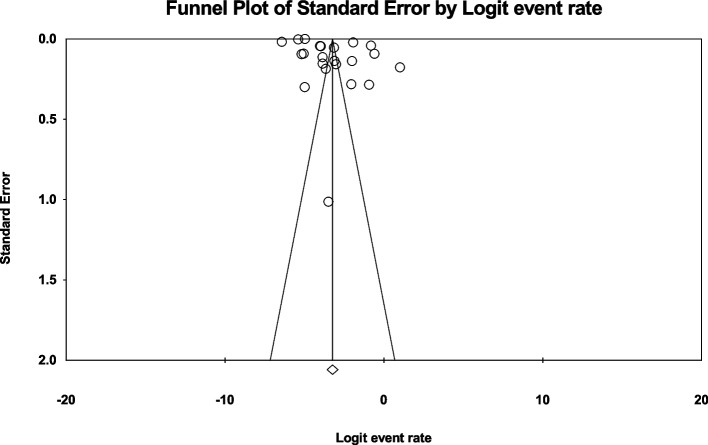
Funnel plot representing publication bias in eligible collected studies

Data analysis of 20 eligible studies with a sample size of 5.071.185 individuals > 60 years was conducted, and *I*^*2*^ index represented a high heterogeneity rate (*I*^*2*^ = 99.7%). Meta-analysis assessment revealed that the global prevalence of MI in individuals > 60 years was 9.5% (95%CI: 7.7–11.6) (Fig. 2). Also, no publication bias (*p* = 0.113) was found in this age group (Fig. 3). Following data analysis of 22 eligible studies with a sample size of 29.826.717 individuals < 60 years, the *I*^*2*^ index showed a high heterogeneity rate (*I*^*2*^ = 99.9). The global MI prevalence in this age group was found 3.8% (95%CI:2.7–5.3) (Fig. 4). Also, no publication bias (*p* = 0.064) was detected (Fig. 5).

## Discussion

This systematic review and meta-analysis study in the first investigation examine the global prevalence of MI in two groups of individuals < 60 and > 60 years. The global prevalence of MI < 60 years was detected 3.8% according to 22 studies with a sample size of 29.826.717 individuals. This value was also found 9.5% in the remaining 20 studies with a sample size of 5.071.185 patients > 60 years.

Following gender categorization, the prevalence of MI in males was found almost 5 folds greater than the females [44]. In a large number of other published studies, a high prevalence of MI was reported in males (> 60%) compared to females [56–79]. On the contrary, other literature reported higher MI prevalence in females, probably due to the sedentary lifestyle, metabolic syndrome, and similar risk factors [80].

Based on the geographical distribution, there were different results representing the MI prevalence including 10.4%, 0.1%, 0.2%, and 2.5% in Sudan, Senegal, Nigeria, and Kenya, respectively. These geographical differences in MI prevalence were probably associated with lifestyle, disease prevention plans, and the level of availability of medical diagnosis resources [81–84].

Extracted data from a large, diverse, community-based population represented a considerable decrease in MI prevalence (after 2000) and incidence of ST-segment elevation (in recent decades) [2]. Although the statistical analysis of CAD prevalence and the related mortality rate showed a decremental trend, the statistics of published literature (before 2002) had no report [85–92].

Various studies conducted in the United States (after 2000) revealed a considerable decremental trend in the incidence of AMI and the rate of hospitalization [2, 93]. The rate of AMI incidence also decreased in Sweden between 2001 to 2008 which was higher in males [94]. A similar trend conducted in the Netherlands from 1998 to 2007 also reported the same results [95]. Respectively, 33% and 31% reduction of AMI rates in males and females were reported in England (2002 to 2010) [96]. Another study showed a steady decline in AMI and mortality rates in most regions of Europe [10]. This study was consistent with the findings of the present investigation reporting that a reduction in MI prevalence was probably associated with innovation of preventive medical protocols and a parallel improvement in risk factors management [95, 97–99].

The prevalence of angina and MI decreased considerably over the 12-year period. The reduction in the prevalence of cardiovascular diseases (CVD), including angina and MI, may result from application of preventive medical procedures and management of risk factors [45]. On the contrary, a high prevalence of undiagnosed MI (26.9%) was also reported. Consequently, more participants (17%) had un-diagnosed MI, and others (9.6%) represented diagnosed MI [47]. In another study, the incidence of definitive MI diagnosis in hospitalized patients was 272/100,000 individuals (aged 30–74) [87].

The high rate and increased severity of CAD in patients with a family background were directly related to the risk of MI in younger ages and both genders [40]. The scientists also found that cocaine addicts are 7 times more at risk of heart attack [100]. Notably, an increased rate of MI incidence was detected in people < 55 years during 1997–2005 [101]. In parallel, various studies reported an annual increase (4%) in the incidence of AMI among women aged 35 to 54 years in Western Australia (from 1996 to 2007) and an increase among women aged 20 to 49 years (from 1994 to 2004). In these studies, the accelerated prevalence of smoking (especially among young females), obesity, and the lack of physical activity have been reported in adolescents and young adults [102–108].

In this study, a higher prevalence was reported in people over the age of 60. In the results reported in the global epidemiology study of ischemic heart disease, which was based on the results of the global burden of disease study, it was reported that ischemic heart disease has a high upward trend with It shows increasing age and the growing trend continues until the age of 89 [109].

### Limitations

Since the age range explained in published studies had no similarity to the age groups in the present study, some eligible papers were excluded. Although, almost half of the studies were conducted in specific subpopulations (such as other heart disease and diabetic patients admitted to the emergency department); difficult conclusions regarding the MI prevalence in general population were possible.

## Conclusion

According to the findings of the present study, the prevalence of MI in people < 60 and > 60 years old were 3.8% and 9.5%, respectively. Therefore, based on the results of the studies that have been reviewed and included in the meta-analysis, the high prevalence of MI was reported to be higher in individuals > 60 years which is considered a warning for health policymakers regarding the importance of this age for diagnosis and screening procedures of MI.

## Data Availability

Datasets are available through the corresponding author upon reasonable request.

## Abbreviations

WoS: Web of Science
PRISMA: Preferred Reporting Items for Systematic Reviews and Meta-Analysis
STROBE: Strengthening the reporting of observational studies in epidemiology for cross-sectional study
MI: Myocardial infarction

## References

[1] Hahla MS, Saeed Y, Razieh H (2016). Comparison of risk factors & clinical and angiographic characterization of STEMI in young adults with older patients. Res J Pharm Biol Chem Sci.

[2] Yeh RW (2010). Population trends in the incidence and outcomes of acute myocardial infarction. N Engl J Med.

[3] Thygesen K (2007). Universal definition of myocardial infarction. J Am Coll Cardiol.

[4] Fox KAA (2007). Decline in rates of death and heart failure in acute coronary syndromes, 1999–2006. J Am Med Assoc.

[5] Goldberg RJ (2001). Twenty-two year (1975 to 1997) trends in the incidence, in-hospital and long-term case fatality rates from initial Q-wave. J Am Coll Cardiol.

[6] Mandelzweig L (2006). The second Euro Heart Survey on acute coronary syndromes: characteristics, treatment, and outcome of patients with ACS in Europe and the Mediterranean Basin in 2004. Eur Heart J.

[7] Liew R (2006). Declining case fatality rates for acute myocardial infarction in South Asian and white patients in the past 15 years. Heart.

[8] Dyrbuś K (2019). The prevalence and management of familial hypercholesterolemia in patients with acute coronary syndrome in the Polish tertiary centre: results from the TERCET registry with 19,781 individuals. Atherosclerosis.

[9] Gerber Y (2015). The changing epidemiology of myocardial infarction in Olmsted County, Minnesota, 1995–2012. Am J Med.

[10] Dégano IR (2015). Twenty-five-year trends in myocardial infarction attack and mortality rates, and case-fatality, in six European populations. Heart.

[11] Moraes-Silva IC, Rodrigues B, Coelho-Junior HJ, Jardim Feriani D, Irigoyen MC. Myocardial Infarction and Exercise Training: Evidence from Basic Science. Adv Exp Med Biol. 2017;999:139–53. 10.1007/978-981-10-4307-9_9 29022262

[12] Velagaleti RS (2008). Long-term trends in the incidence of heart failure after myocardial infarction. Circulation.

[13] Juilliere Y (2012). Heart failure in acute myocardial infarction: a comparison between patients with or without heart failure criteria from the FAST-MI registry. Revista Española de Cardiología (English Edition).

[14] Lewis EF (2003). Predictors of late development of heart failure in stable survivors of myocardial infarction: the CARE study. J Am Coll Cardiol.

[15] Members WG (2012). Heart disease and stroke statistics—2012 update: a report from the American Heart Association. Circulation.

[16] Weintraub WS (2011). Value of primordial and primary prevention for cardiovascular disease: a policy statement from the American Heart Association. Circulation.

[17] Fried LP (2001). Frailty in older adults: evidence for a phenotype. J Gerontol A Biol Sci Med Sci.

[18] Zarich S (2006). Prevalence of metabolic syndrome in young patients with acute MI: does the Framingham risk score underestimate cardiovascular risk in this population?. Diab Vasc Dis Res.

[19] Puymirat E (2012). Association of changes in clinical characteristics and management with improvement in survival among patients with ST-elevation myocardial infarction. JAMA.

[20] Chockalingam A, Campbell NR, Fodor JG (2006). Worldwide epidemic of hypertension. Can J Cardiol.

[21] Chung Chooi Y, Ding C, Magkos F. The epidemiology of obesity. Metabolism. 2019; 92:6–10. 10.1016/j.metabol.2018.09.005 30253139

[22] Vähätalo JH, Huikuri HV, Holmström LTA, Kenttä TV, Haukilahti MAE, Pakanen L. Association of Silent Myocardial Infarction and Sudden Cardiac Death. JAMA Cardiol. 2019 1;4(8):796–802. 10.1001/jamacardio.2019.2210 PMC6624824 31290935

[23] Marques-Vidal P (2001). Distribution and treatment of cardiovascular risk factors in coronary patients: the Prevenir Study. Arch Mal Coeur Vaiss.

[24] Chow CM (2005). Regional variation in self-reported heart disease prevalence in Canada. Can J Cardiol.

[25] Assante R (2015). Prevalence and severity of myocardial perfusion imaging abnormalities in inmate subjects. PLoS ONE.

[26] Carrillo X (2011). Acute coronary syndrome and cocaine use: 8-year prevalence and inhospital outcomes. Eur Heart J.

[27] Bosch X (2010). Prevalence, clinical characteristics and risk of myocardial infarction in patients with cocaine-related chest pain. Rev Esp Cardiol.

[28] Bulow B (2000). Hypopituitary females have a high incidence of cardiovascular morbidity and an increased prevalence of cardiovascular risk factors. J Clin Endocrinol Metab.

[29] Chung EH (2007). Prevalence of metabolic syndrome in patients < or =45 years of age with acute myocardial infarction having percutaneous coronary intervention. Am J Cardiol.

[30] Domingos F, Serra A (2011). Nephrolithiasis is associated with an increased prevalence of cardiovascular disease. Nephrol Dial Transplant.

[31] Gikas A (2008). Prevalence trends for myocardial infarction and conventional risk factors among Greek adults (2002–06). QJM.

[32] Gisondi P, DalleVedove C, Girolomoni G (2011). Patients with psoriasis have a higher prevalence of parental cardiovascular disease. Dermatology.

[33] Ingelfinger JA (1976). Coronary heart disease in the Pima Indians. Electrocardiographic findings and postmortem evidence of myocardial infarction in a population with a high prevalence of diabetes mellitus. Diabetes.

[34] Khan H (2022). Gender differences in prevalence of myocardial infarction in rural West Texans. J Public Health-Heidelberg.

[35] Kitamura A (2002). Trends in the incidence of coronary heart disease and stroke and the prevalence of cardiovascular risk factors among Japanese men from 1963 to 1994. Am J Med.

[36] Lampe FC (2001). Is the prevalence of coronary heart disease falling in British men?. Heart.

[37] Lautsch D (2019). Prevalence of established cardiovascular disease in patients with type 2 diabetes mellitus in the UK. Diabetes Therapy.

[38] McCullough PA (2008). Chronic kidney disease, prevalence of premature cardiovascular disease, and relationship to short-term mortality. Am Heart J.

[39] Okoth K, et al. Sex-specific temporal trends in the incidence and prevalence of cardiovascular disease in young adults: a population-based study using UK primary care data. Eur J Prev Cardiol. 2022;29(10):1387–95. 10.1093/eurjpc/zwac024 35139185

[40] Otaki Y (2013). Impact of family history of coronary artery disease in young individuals (from the CONFIRM registry). Am J Cardiol.

[41] Sato K (2020). Temporal trends in the prevalence and outcomes of geriatric patients with acute myocardial infarction in Japan–a report from the Miyagi AMI Registry Study–. J Cardiol.

[42] Shaper AG (1984). Prevalence of ischaemic heart disease in middle aged British men. Br Heart J.

[43] Zeller T (2014). High population prevalence of cardiac troponin I measured by a high-sensitivity assay and cardiovascular risk estimation: the MORGAM Biomarker Project Scottish Cohort. Eur Heart J.

[44] Zeidan RK (2016). Prevalence and correlates of coronary heart disease: first population-based study in Lebanon. Vasc Health Risk Manag.

[45] Yoon SSS (2016). Trends in the prevalence of coronary heart disease in the US: National Health and Nutrition Examination Survey, 2001–2012. Am J Prev Med.

[46] Valentine RJ (1994). Coronary artery disease is highly prevalent among patients with premature peripheral vascular disease. J Vasc Surg.

[47] Schelbert EB (2012). Prevalence and prognosis of unrecognized myocardial infarction determined by cardiac magnetic resonance in older adults. JAMA.

[48] Kumar A (2008). Prevalence, prognosis, and implications of isolated minor nonspecific ST-segment and T-wave abnormalities in older adults cardiovascular health study. Circulation.

[49] Bahrmann P (2013). A 3-hour diagnostic algorithm for non-ST-elevation myocardial infarction using high-sensitivity cardiac troponin T in unselected older patients presenting to the emergency department. J Am Med Dir Assoc.

[50] Bethel MA (2017). Assessing the safety of sitagliptin in older participants in the Trial Evaluating Cardiovascular Outcomes with Sitagliptin (TECOS). Diabetes Care.

[51] Cauley JA (2016). Risk factors for hip fracture in older men: the osteoporotic fractures in men study (MrOS). J Bone Miner Res.

[52] de la Torre Hernández JM (2017). Primary angioplasty in patients older than 75 years profile of patients and procedures, outcomes, and predictors of prognosis in the ESTROFA IM+75 registry. Rev Esp Cardiol (Engl Ed).

[53] Golledge J (2014). Reported high salt intake is associated with increased prevalence of abdominal aortic aneurysm and larger aortic diameter in older men. PLoS ONE.

[54] Ikeda Y (2014). Low-dose aspirin for primary prevention of cardiovascular events in Japanese patients 60 years or older with atherosclerotic risk factors: a randomized clinical trial. JAMA.

[55] Teo KK (2009). Optimal medical therapy with or without percutaneous coronary intervention in older patients with stable coronary disease: a pre-specified subset analysis of the COURAGE (Clinical Outcomes Utilizing Revascularization and Aggressive druG Evaluation) trial. J Am Coll Cardiol.

[56] Dixon WC (2008). Anatomic distribution of the culprit lesion in patients with non–ST-segment elevation myocardial infarction undergoing percutaneous coronary intervention: findings from the National Cardiovascular Data Registry. J Am Coll Cardiol.

[57] Kim MC (2012). Impact of total occlusion of an infarct-related artery on long-term mortality in acute non-ST-elevation myocardial infarction patients who underwent early percutaneous coronary intervention. Int Heart J.

[58] Shin DI (2014). Impact of occluded culprit arteries on long-t erm clinical outcome in patients with non-ST-elevation myocardial infarction: 48-month follow-u p results in the COREA-AMI registry. J Interv Cardiol.

[59] Jung DH (2008). Predictors of total occlusion of the infarct-related artery in patients with acute non-ST elevation myocardial infarction. Korean J Med.

[60] Pride YB (2010). Angiographic and clinical outcomes among patients with acute coronary syndromes presenting with isolated anterior ST-segment depression: a TRITON–TIMI 38 (trial to assess improvement in therapeutic outcomes by optimizing platelet inhibition with Prasugrel–thrombolysis In myocardial infarction 38) substudy. JACC Cardiovasc Interv.

[61] Bahrmann P (2011). Incidence and distribution of occluded culprit arteries and impact of coronary collaterals on outcome in patients with non-ST-segment elevation myocardial infarction and early invasive treatment strategy. Clin Res Cardiol.

[62] Warren J (2015). Incidence and impact of totally occluded culprit coronary arteries in patients presenting with non–ST-segment elevation myocardial infarction. Am J Cardiol.

[63] Yazici M, Demircan S, Durna K (2007). Association between nitric oxide levels on myocardial injury in non-ST elevation acute coronary syndromes. J Thromb Thrombolysis.

[64] Abbott JD (2007). Comparison of outcome in patients with ST-elevation versus non–ST-elevation acute myocardial infarction treated with percutaneous coronary intervention (from The National Heart, Lung, and Blood Institute Dynamic Registry). Am J Cardiol.

[65] Karwowski J (2016). Post-procedural TIMI flow grade 2 is not associated with improved prognosis in patients with non-ST-segment elevation myocardial infarction undergoing percutaneous coronary revascularization (PL-ACS registry). Cardiol J.

[66] Soon K (2014). Non-ST elevation myocardial infarction with occluded artery and its clinical implications. Heart Lung Circ.

[67] Aijaz S, Hanif B (2016). Frequency and distribution of angiographically occluded coronary artery and in-hospital outcome of patients with Non ST elevation myocardial infarction. J Pak Med Assoc.

[68] Daly M (2012). Detection of acute coronary occlusion in patients with acute coronary syndromes presenting with isolated ST-segment depression. Eur Heart J Acute Cardiovasc Care.

[69] Wong GC (2002). Elevations in troponin T and I are associated with abnormal tissue level perfusion: a TACTICS-TIMI 18 substudy. Circulation.

[70] Koyama Y (2002). Prevalence of coronary occlusion and outcome of an immediate invasive strategy in suspected acute myocardial infarction with and without ST-segment elevation. Am J Cardiol.

[71] Bolognese L (2004). Elevations in troponin I after percutaneous coronary interventions are associated with abnormal tissue-level perfusion in high-risk patients with non–ST-segment–elevation acute coronary syndromes. Circulation.

[72] Abbas AE (2004). Acute angiographic analysis of non–ST-segment elevation acute myocardial infarction. Am J Cardiol.

[73] Mazurek M (2011). The impact of unsuccessful percutaneous coronary intervention on short-and long-term prognosis in STEMI and NSTEMI. Catheter Cardiovasc Interv.

[74] Widimsky P (2012). Primary angioplasty in acute myocardial infarction with right bundle branch block: should new onset right bundle branch block be added to future guidelines as an indication for reperfusion therapy?. Eur Heart J.

[75] Park H-W (2013). Early-and late-term clinical outcome and their predictors in patients with ST-segment elevation myocardial infarction and non-ST-segment elevation myocardial infarction. Int J Cardiol.

[76] Zhang D (2014). The effects of tirofiban on acute non-ST segment elevation myocardial infarction patients not receiving early reperfusion intervention. Zhonghua Nei Ke Za Zhi.

[77] Guerra E (2014). Microvascular obstruction in patients with non-ST-elevation myocardial infarction: a contrast-enhanced cardiac magnetic resonance study. Int J Cardiovasc Imaging.

[78] Liu N (2015). Clinical research of treatment with tirofiban for high-risk non-ST-segment elevation acute coronary syndrome during peri-operative intervention operation period. Cell Biochem Biophys.

[79] Misumida N (2015). Association between preinfarction angina and angiographic findings in non–ST-segment elevation myocardial infarction. Clin Cardiol.

[80] Kastrati A (2011). Abciximab and heparin versus bivalirudin for non–ST-elevation myocardial infarction. N Engl J Med.

[81] Seck M (2007). Profile of patients admitted for myocardial infarction at the emergency reception facility of Principal Hospital in Dakar, Senegal. Med Trop.

[82] Kolo PM (2013). Changing trend in the incidence of myocardial infarction among medical admissions in Ilorin, north-central Nigeria. Niger Postgrad Med J.

[83] Sani M (2006). Ischaemic heart disease in Aminu Kano teaching hospital, Kano, Nigeria: a 5 year review. Niger J Med.

[84] Nguchu H, Joshi M, Otieno C (2009). Acute coronary syndromes amongst type 2 diabetics with ischaemic electrocardiograms presenting to accident and emergency department of a Kenyan tertiary institution. East Afr Med J.

[85] Sytkowski PA (1996). Sex and time trends in cardiovascular disease incidence and mortality: the Framingham Heart Study, 1950–1989. Am J Epidemiol.

[86] Fox CS (2004). Temporal trends in coronary heart disease mortality and sudden cardiac death from 1950 to 1999: the Framingham Heart Study. Circulation.

[87] McGovern PG (2001). Trends in acute coronary heart disease mortality, morbidity, and medical care from 1985 through 1997: the Minnesota heart survey. Circulation.

[88] Goldberg RJ (2004). A 25-year perspective into the changing landscape of patients hospitalized with acute myocardial infarction (the Worcester Heart Attack Study). Am J Cardiol.

[89] Arciero TJ (2004). Temporal trends in the incidence of coronary disease. Am J Med.

[90] Roger VL (2002). Trends in the incidence and survival of patients with hospitalized myocardial infarction, Olmsted County, Minnesota, 1979 to 1994. Ann Intern Med.

[91] Masoudi FA (2006). Trends in acute myocardial infarction in 4 US states between 1992 and 2001: clinical characteristics, quality of care, and outcomes. Circulation.

[92] Parikh NI (2009). Long-term trends in myocardial infarction incidence and case fatality in the National Heart, Lung, and Blood Institute’s Framingham Heart study. Circulation.

[93] Chen J (2010). Recent declines in hospitalizations for acute myocardial infarction for medicare fee-for-service beneficiaries: progress and continuing challenges. Circulation.

[94] Yang D (2012). Incidence and case fatality after day 28 of first time myocardial infarction in Sweden 1987–2008. Eur J Prev Cardiol.

[95] Koopman C (2013). Population trends and inequalities in incidence and short-term outcome of acute myocardial infarction between 1998 and 2007. Int J Cardiol.

[96] Smolina K (2012). Determinants of the decline in mortality from acute myocardial infarction in England between 2002 and 2010: linked national database study. BMJ.

[97] Lundblad D (2008). Gender differences in trends of acute myocardial infarction events: the Northern Sweden MONICA study 1985–2004. BMC Cardiovasc Disord.

[98] van Oeffelen AAM (2014). Downward trends in acute myocardial infarction incidence: how do migrants fare with the majority population? Results from a nationwide study. Eur J Prev Cardiol.

[99] Sulo G (2014). Favourable trends in incidence of AMI in Norway during 2001–2009 do not include younger adults: a CVDNOR project. Eur J Prev Cardiol.

[100] Aslibekyan S, Levitan EB, Mittleman MA (2008). Prevalent cocaine use and myocardial infarction. Am J Cardiol.

[101] McManus DD (2011). Thirty-year (1975 to 2005) trends in the incidence rates, clinical features, treatment practices, and short-term outcomes of patients< 55 years of age hospitalized with an initial acute myocardial infarction. Am J Cardiol.

[102] Egred M, Viswanathan G, Davis G (2005). Myocardial infarction in young adults. Postgrad Med J.

[103] Briffa TG (2011). Population trends of recurrent coronary heart disease event rates remain high. Circ Cardiovasc Qual Outcomes.

[104] Clench-Aas J, Helgeland J, Dimoski T, Gulbrandsen P, Hofoss D, Holmboe O, et al. Methodological Development and Evaluation of 30-Day Mortality as Quality Indicator for Norwegian Hospitals [Internet]. Oslo, Norway: Knowledge Centre for the Health Services at The Norwegian Institute of Public Health (NIPH); 2005 . Report from Norwegian Knowledge Centre for the Health Services (NOKC) No. 04-2005. 29319970

[105] Ahmad OB (2001). Age standardization of rates: a new WHO standard. Geneva: World Health Organization.

[106] Schmidt M (2012). 25 year trends in first time hospitalisation for acute myocardial infarction, subsequent short and long term mortality, and the prognostic impact of sex and comorbidity: a Danish nationwide cohort study. BMJ.

[107] Luepker RV. Decline in incident coronary heart disease: why are the rates falling? 2008, Circulation. 2008;117(5):592–3. 10.1161/CIRCULATIONAHA.107.747477 18250277

[108] Tu JV (2009). National trends in rates of death and hospital admissions related to acute myocardial infarction, heart failure and stroke, 1994–2004. CMAJ.

[109] Khan MA, Hashim MJ, Mustafa H, Baniyas MY, Buti Mohamad Al Suwaidi SK, AlKatheeri R (2020). Global epidemiology of ischemic heart disease: results from the global burden of disease study. Cureus.

